# Proceedings of the International Workshop ‘From Global Burden of Disease Studies to National Burden of Disease Surveillance': Berlin, Germany. 1-2 December 2015

**DOI:** 10.1186/s12919-016-0005-1

**Published:** 2016-07-25

**Authors:** Christa Scheidt-Nave, Thomas Ziese, Judith Fuchs, Dietrich Plass, Tom Achoki, Katherine Leach-Kemon, Peter Speyer, William E. Heisel, Emmanuela Gakidou, Theo Vos, Mohammad Hossein Forouzanfar, Jürgen C. Schmidt, Claudia E. Stein, Christa Scheidt-Nave, Elena von der Lippe, Benjamin Barnes, Markus A. Busch, Nina Buttmann-Schweiger, Judith Fuchs, Christin Heidemann, Klaus Kraywinkel, Enno Nowossadeck, Thomas Ziese, Udo Buchholz, Matthias an der Heiden, Tim Eckmanns, Sebastian Haller, Mohammad Hossein Forouzanfar, Dietrich Plass, Myriam Tobollik, Dagmar Kallweit, Dirk Wintermeyer, Christa Scheidt-Nave, Thomas Ziese, Judith Fuchs, Dietrich Plass

**Affiliations:** 1 Department of Epidemiology and Health Monitoring, Robert Koch Institute, Berlin, Germany; 2 German Environment Agency, Berlin, Germany; 3 Institute for Health Metrics and Evaluation, Department of Global Health, University of Washington, Seattle, Washington USA; 4 Institute for Health Metrics and Evaluation (IHME), Seattle, USA; 5 Public Health England, London, SE1 6LH United Kingdom; 6 Division of Information, Evidence, Research and Innovation, World Health Organization Regional Office for Europe, Copenhagen, Denmark; 7 Department of Epidemiology and Health Monitoring, Robert Koch Institute, Berlin, Germany; 8 Department for Infectious Disease Epidemiology, Robert Koch Institute, Berlin, Germany; 9 Institute for Health Metrics and Evaluation (IHME), Seattle, USA; 10 German Environment Agency, Berlin, Germany; 11 Department of Epidemiology and Health Monitoring, Robert Koch Institute, Berlin, Germany; 12 German Environment Agency, Berlin, Germany

## Abstract

I1 Introduction and aims of the workshop

*Christa Scheidt-Nave*, Thomas Ziese, Judith Fuchs, Dietrich Plass

S1 History, concept, and current results of GBD for Germany

Tom Achoki, Katherine Leach-Kemon, Peter Speyer, William E. Heisel, Emmanuela Gakidou, Theo Vos

S2 Methodology of the GBD 2013 Study–Mortality, Morbidity, Risk-Factors

Mohammad Hossein Forouzanfar

S3 National burden of disease surveillance examples of good practice: the case of Public Health England

Jürgen C. Schmidt

S4 Critical aspects of the burden of disease methodology and country-specific challenges

Claudia E. Stein

S5 Non-communicable disease surveillance in Germany – public health and data challenges

Christa Scheidt-Nave, Elena von der Lippe, Benjamin Barnes, Markus Busch, Nina Buttmann-Schweiger, Judith Fuchs, Christin Heidemann, Klaus Kraywinkel, Enno Nowossadeck, Thomas Ziese

S6 Different approaches in estimating the burden of communicable diseases using the examples of the healthcare associated infections and influenza

Udo Buchholz, Matthias an der Heiden, Tim Eckmanns, Sebastian Haller

S7 Behavioral and environmental attributable risk estimation

Mohammad Hossein Forouzanfar

S8 Environmental Burden of Disease (EBD) in Germany – past achievements and future perspectives

Dietrich Plass, Myriam Tobollik, Dagmar Kallweit, Dirk Wintermeyer

C1 Conclusions of the workshop

Christa Scheidt-Nave, Thomas Ziese, Judith Fuchs, Dietrich Plass

## I1 Introduction and aims of the workshop

### Christa Scheidt-Nave^1^, Thomas Ziese^1^, Judith Fuchs^1^, Dietrich Plass^2^

#### ^1^Department of Epidemiology and Health Monitoring, Robert Koch Institute, Berlin, Germany; ^2^German Environment Agency, Berlin, Germany

##### **Correspondence:** Christa Scheidt-Nave (Scheidt-NaveC@rki.de) – Department of Epidemiology and Health Monitoring, Robert Koch Institute, Berlin, Germany

The Global Burden of Disease (GBD) framework was jointly developed by the WHO, the World Bank and the Harvard School of Public Health in the early 1990s with the aim to permit a first comprehensive and comparable evaluation of population health for all countries of the world [1]. The possibility to compare disease burden over time and between countries provides valuable insights into intervention successes, failures as well as unfinished agendas and can also highlight emerging health threats. Since the first GBD analyses several updates of the GBD study were performed by the WHO.

The Institute for Health Metrics and Evaluation (IHME) has recently presented a fully refreshed set of global disease burden estimates and aims for updates on a regular basis [2]. To provide information that is useful to guide public health policy decision-making processes at the national level, IHME researchers, WHO and public health researchers from individual countries need to intensify their dialogue on research demands, the necessary methods, and the quality of the underlying data used for such assessments. To this effect, WHO and IHME recently signed a memorandum of understanding for enhanced collaboration and data sharing.

The international workshop “From Global Burden of Disease Studies to National Burden of Disease Surveillance” aimed to enhance the cooperation in public health research and exchange of information between researchers of the GBD study and public health and environmental health institutes in Germany. The framework, methodology and results from recent international and national GBD analyses were presented by researchers from the IHME and Public Health England. The Workshop was organized by the Department of Epidemiology and Health Monitoring at Germany’s Robert Koch Institute (RKI) and the German Environment Agency (UBA) in cooperation with the German Society for Epidemiology (DGEpi) e.V. and Bielefeld University.

Maria Krautzberger, president of the UBA and Prof. Dr. Lothar Wieler, president of the RKI emphasized in their welcome addresses the importance for a strong collaboration on burden of disease analyses between the WHO, the IHME, and the public health research network in Germany including RKI, UBA as well as representatives from various public health and epidemiology departments and professional societies.

During two days, a team of scientists from international and German public organizations discussed GBD methods and results as well as strategies for national burden of disease analyses with an audience of over 60 attendees. In total, 8 presentations followed by intensive discussions were delivered.

**References**

1. GBD 2013 DALYs and HALE Collaborators (2015) Global, regional, and national disability-adjusted life years (DALYs) for 306 diseases and injuries and healthy life expectancy (HALE) for 188 countries, 1990-2013: quantifying the epidemiological transition. Lancet 386(10009): 2145-2191.

2. Murray CJL, Lopez AD (1996). The Global Burden of Disease: A comprehensive assessment of mortality and disability from diseases, injuries, and risk factors in 1990 and projected to 2020. Cambridge, Harvard School of Public Health on behalf of the World Health Organization and the World Bank.

## S1 History, concept, and current results of GBD for Germany

### Tom Achoki, Katherine Leach-Kemon, Peter Speyer, William E. Heisel, Emmanuela Gakidou, Theo Vos

#### Institute for Health Metrics and Evaluation, Department of Global Health, University of Washington, Seattle, Washington, USA

##### **Correspondence:** Tom Achoki (tachoki@uw.edu) – Institute for Health Metrics and Evaluation, Department of Global Health, University of Washington, Seattle, Washington, USA

The Global Burden of Disease (GBD) quantifies and compares health loss due to diseases, injuries, and risk factors by age, sex, and geography over time. The latest iteration of the GBD 2013, was published in The Lancet in 2014 and 2015 [1-4]. As a global public good, GBD is useful for informing the design of health systems and the creation of public health policy. It estimates premature death and disability due to 306 diseases and injuries, 2,337 sequelae, and 79 risk factors by age and sex for 1990, 1995, 2000, 2005, 2010, and 2013. GBD 2013 produced estimates for 188 countries. This is a collaborative effort among more than 1,500 researchers from 120 countries, and the Institute for Health Metrics and Evaluation (IHME) is the coordinating center for the study.

The first GBD study was published as part of the World Development Report 1993. The authors’ inspiration for the study came from realizing that policymakers lacked sound, comprehensive, and standardized data on diseases, injuries, and potentially preventable risk factors for decision-making. The GBD 1990 study had a profound impact on health policy as it exposed the hidden burden of mental illness around the world and shed light on neglected health areas such as road traffic injuries.

According to GBD 2013 results, life expectancy in Germany increased by 4.6 years for females and 6.2 years for males between 1990 and 2013. Low back and neck pain was the leading cause of disability-adjusted life years (DALYs), with ischemic heart disease and stroke being the second- and third-leading causes, respectively. Disability-adjusted life years include health loss due to premature death and disability. Total DALYs from both ischemic heart disease and stroke decreased by more than 30 % from 1990 to 2013. The leading risk factor for DALYs in 2013 was dietary risks, accounting for more than 25 % of total DALYs. High systolic blood pressure and high body mass index were the second- and third-leading risk factors for DALYs in 2013, respectively. Benchmarking Germany’s performance for leading causes of premature death against peer countries reveals that Germany performed significantly worse for ischemic heart disease but significantly better for chronic obstructive pulmonary disease and road injuries. Considering that aggregate estimates often conceal important subnational differences, collaborative efforts towards understanding the subnational burden of disease, would be more useful for Germany policy making.

**References**

1. GBD 2013 Mortality and Causes of Death Collaborators (2015) Global, regional, and national age-sex specific all-cause and cause-specific mortality for 240 causes of death, 1990–2013: a systematic analysis for the Global Burden of Disease Study 2013.385(9963): 117–171.

2. Global Burden of Disease Study 2013 Collaborators (2015) Global, regional, and national incidence, prevalence, and years lived with disability for 301 acute and chronic diseases and injuries in 188 countries, 1990–2013: a systematic analysis for the Global Burden of Disease Study 2013. Lancet. 386(9995):743–800.

3. GBD 2013 DALYs and HALE Collaborators (2015) Global, regional, and national disability-adjusted life years (DALYs) for 306 diseases and injuries and healthy life expectancy (HALE) for 188 countries, 1990-2013: quantifying the epidemiological transition. Lancet. 386(10009): 2145-2191.

4. GBD 2013 Risk Factors Collaborators (2015) Global, regional, and national comparative risk assessment of 79 behavioral, environmental and occupational, and metabolic risks or clusters of risks in 188 countries, 1990–2013: a systematic analysis for the Global Burden of Disease Study 2013. Lancet. 386(10010):2287–2323.

## S2 Methodology of the GBD 2013 Study–Mortality, Morbidity, Risk-Factors

### Mohammad Hossein Forouzanfar (forouzan@uw.edu)

#### Institute for Health Metrics and Evaluation (IHME), Seattle/USA

The Global Burden of Diseases, Injuries and Risk Factors study 2013 (GBD) is the second update to a series of studies initiated by the World Bank, the Harvard School of Public Health and WHO in the 1990s. The main aims of the first GBD study were to present the health status of the global population and to introduce a methodology for a comprehensive and comparable assessment of the global disease burden including all relevant entities causing ill-health. The first GBD assessment highlighted the importance of mental conditions and injuries for the global health status and the recent updates strongly emphasized the impact of the global epidemiological and demographic transition on population health. One of the central measures introduced by the GBD framework is the Disability-Adjusted Life Year (DALY) which estimates healthy life years lost due to the effects of mortality and morbidity. This measure allows the quantification of health losses comparable between different health conditions, population groups and over time. The current GBD assessments include four major groups of efforts including: 1) estimating all-cause mortality and life expectancy, 2) cause-specific mortality, 3) morbidity and disability weight estimation, and 4) covariates and risk factor burden estimation. The GBD 2013 involved the collection and analysis of global health data to estimate the burden of diseases measured by DALY and life expectancy as well as healthy life expectancy (HALE) for 188 countries, China, Mexico and UK subnational areas, for 20 age-groups, both sexes, and for each 5 year interval for the years 1990 – 2010 and for 2013 [1]. Different data sources as well as cause- and risk-specific methods and analytical tools were employed to deal with data scarcity and estimate the health outcomes and risk factor burden. The governance model for GBD is designed to incorporate the expertise of more than 1500 collaborators. The GBD is strengthened by a rich scientific team, a strong computational infrastructure and visualization experts, as well as teams specializing in disseminating and engaging policy-makers, government agencies, and others in the use of the results. In this presentation different GBD 2013 sections, outcome measures, causes as well as the structure of sequelae and risk factor estimation are explained. Further, major methods such as CODEm [2], DisMod-MR and challenges such as garbage code redistribution and risk factor aggregation were discussed. The presentation also gave an introduction to the GBD visualization tool-suite [3].

**References**

1. GBD 2013 DALYs and HALE Collaborators (2015) Global, regional, and national disability-adjusted life years (DALYs) for 306 diseases and injuries and healthy life expectancy (HALE) for 188 countries, 1990-2013: quantifying the epidemiological transition. Lancet. 386(10009): 2145-2191.

2. Foreman KJ, Lozano R, Lopez AD, Murray CJ (2012) Modeling causes of death: an integrated approach using CODEm." Population Health Metrics 10: 1.

3. Institute for Health Metrics and Evaluation (2016) http://www.healthdata.org/gbd/data-visualizations [Accessed 31 May 2016]

## S3 National burden of disease surveillance examples of good practice: the case of Public Health England

### Jürgen C. Schmidt (Jurgen.Schmidt@phe.gov.uk)

#### Public Health England, London SE1 6LH, United Kingdom

The England Burden of Disease programme builds upon the GBD (Global Burden of Disease) experience in order to

- present an England as opposed to a UK perspective
- provide quality assurance of English data used while exercising version control
- allow a subnational breakdown of analysis
- investigate links between risk factors, deprivation, geography, and outcomes

Data going back up to 20 years were provided to the Institute for Health Metrics and Evaluation on mortality, morbidity and risk factors. These were broken down by nine English regions, corresponding to the former Government Office Regions in England. The Index of Multiple Deprivation (IMD-2010) was used, which allocates a poverty score to each LSOA (lower super output area) in each region. LSOAs are relatively homogeneous areas containing about 1600 people on average. The IMD-2010 is a composite measure estimated at a small geographical area and includes seven domains: income, employment, health and disability, education skills and training, barriers to housing and services, living environment, and crime. Combining information about regions and LSOAs allowed creating 45 regional deprivation areas in total.

Before this exercise no single accessible source describing overall disease burden by cause existed for England. The subsequent analysis [1] provides policy-makers with important headline results:

- England performs above average vs other high income countries on key health outcomes, with life expectancy from birth increased by more than 5 years between 1990 and 2013
- Gains are greater for men than for women
- Large improvements in rates of premature mortality but not in morbidity means living longer but spending more years in ill-health
- Health inequalities (in life expectancy but also in prevalence of preventable diseases) persist, largely driven by deprivation; important within regions as well as between regions
- 40 % of ill health in England is due to potentially preventable risk factors, with unhealthy diet and tobacco being the two biggest risks. This compares unfavourably with the funding provided for prevention.

Current discussions about the future application of the GBD methodology in England examine its use in surveillance, in particular

- as part of the standard suite of tools developed by Public Health England
- for provision of estimates at local level as opposed to national and regional level
- use of GBD outputs as inputs for modelling
- international collaboration with peer countries, especially on non-communicable diseases

**References**

1. Newton JN, Briggs ADM, Murray CJL. Changes in health in England, with analysis by English regions and areas of deprivation, 1990–2013: a systematic analysis for the Global Burden of Disease Study 2013. Lancet. 2015; 386 (10010): 2257 - 2274

2. “The English Indices of Deprivation 2010”, Department for Communities and Local Government, 2011 (available at https://www.gov.uk/government/uploads/system/uploads/attachment_data/file/6871/1871208.pdf

## S4 Critical aspects of the burden of disease methodology and country-specific challenges

### Claudia E. Stein (CLS@euro.who.int)

#### Division of Information, Evidence, Research and Innovation, World Health Organization Regional Office for Europe, Copenhagen, Denmark

The global burden of disease methodology developed by Murray and Lopez [1] has been used by the World Health Organization for over 20 years. The burden of disease approach is very ‘data hungry’ requiring sex- and age-disaggregated information on deaths and diseases, with data sources ranging from vital statistics and civil registration systems, as well as disease registers, hospital discharge data and surveys examining disease and risk factor prevalence. The strengths of the burden approach include the development of a summary measure that combines mortality, morbidity and disability, where years of life lost (YLL) are added to years lived with disability (YLD). It permits the measurement of loss of health in an internationally comparable way, the de-coupling of advocacy from epidemiological assessment and the avoiding of double counting. Numerous countries have conducted national burden of disease studies but frequently struggle with lack of or fragmentation of data at the national level (in which case uncertainty analyses are an important feature), a focus on fatal outcomes and concerns about the appropriateness of disability weights for their own settings. The latter can be a serious obstacle, and some countries have embarked on developing their own disability weights which are seen as more appropriate for the local context but result in reduced international comparability between studies. In some instances, the value judgments associated with disability weights as well as potential age-weighting have even rendered the burden of disease approach as unacceptable to policy makers. In addition, a tendency to ‘start with’ or settle for an abbreviated list of causes instead of the full set of ICD 10 compatible causes is tempting but may lead to skewed results and a masking of the actual burden in the country. In addition, adjustments for co-morbidity are not currently made and the need to allocate one single cause of death is perceived by some as not ‘based in reality’; while this issue remains unresolved, some countries have shied away from the burden approach altogether. The presentation at the workshop discusses these issues and proposes some ways forward for countries wishing to conduct full burden of disease studies. The author calls for the development of a practical manual for national burden of disease studies to guide countries in conducting their national studies by preserving international comparability while at the same time allowing to respect the local context.

**References**

1. Murray CJL, Lopez AD (1996). The Global Burden of Disease: A comprehensive assessment of mortality and disability from diseases, injuries, and risk factors in 1990 and projected to 2020. Cambridge, Harvard School of Public Health on behalf of the World Health Organization and the World Bank.

## S5 Non-communicable disease surveillance in Germany – public health and data challenges

### Christa Scheidt-Nave, Elena von der Lippe, Benjamin Barnes, Markus A. Busch, Nina Buttmann-Schweiger, Judith Fuchs, Christin Heidemann, Klaus Kraywinkel, Enno Nowossadeck, Thomas Ziese

#### Department of Epidemiology and Health Monitoring, Robert Koch Institute, Berlin, Germany

##### **Correspondence:** Christa Scheidt-Nave (Scheidt-NaveC@rki.de) – Department of Epidemiology and Health Monitoring, Robert Koch Institute, Berlin, Germany

As a country in an advanced stage of demographic and epidemiological transition, Germany faces an increasing burden of non-communicable diseases and age-related health conditions [1]. A public health approach to effective health promotion and prevention at all stages of life is needed to delay age-related degenerative diseases and functional decline. While average life expectancy has continuously increased for both sexes in Germany over the past decades, Germany ranks well behind several other European countries. This is especially true for disability-free life expectancy, expressed as the number of remaining years an individual can expect to live without any disability at a defined age (Healthy Life Years indicator). There are large regional differences in life expectancy and in the prevalence of non-communicable diseases and associated risk factors in Germany. These differences are highly correlated with both individual- and area-level socioeconomic differences [2].

To deliver guidance to health politicians and health care providers, Germany is in need of strong public health information systems that permit continuous monitoring of disease burden and the driving causes, including socioeconomic context variables, environmental and behavioral risk factors, and joint risk profiles. In 2008 the Federal Ministry of Health Germany commissioned the Robert Koch Institute to establish a continuous health monitoring system at the national level based on repeated cross-sectional health surveys and a national cohort study of children and adolescents [3-4]. Furthermore, the Centre for Cancer Registry Data (ZfKD) was established at the Robert Koch Institute in 2010, allowing continuous monitoring of time trends in site-specific cancer incidence and mortality based on pooled data from regional population-based cancer registries [5]. A major goal is to further strengthen the national health indicator database by including timely information obtained from sustainable sources of routine health care data, e. g. statutory health insurance data.

National burden of disease analyses based on the Disability-Adjusted Life Year (DALY), a summary measure of population health, may prove useful for public health goal and priority setting. However, this largely depends on the quality and availability of input data. Harmonizing data collection, analysis and interpretation at the national and sub-national level remains a major issue in Germany. A pilot project is currently underway and will focus on improved diabetes surveillance. It will also be necessary to validate disability weights in national health surveys considering not only individual disease states, but also multimorbidity, states of critical functional impairment, potentially inappropriate medication, and avoidable hospital admissions.

**References**

1. Plass D, Vos T, Hornberg C, Scheidt-Nave C, Zeeb H, Krämer A. Trends in disease burden in Germany: results, implications and limitations of the Global Burden of Disease study. Dtsch Arztebl Int. 2014; 111:629-638.

2. Maier W, Scheidt-Nave C, Holle R, Kroll LE, Lampert T, Du Y, Heidemann C, Mielck A. Area level deprivation is an independent determinant of prevalent type 2 diabetes and obesity at the national level in Germany. Results from the National Telephone Health Interview Surveys 'German Health Update' GEDA 2009 and 2010. PLoS One. 2014; 9:e89661. doi: 10.1371/journal.pone.0089661.

3. Heidemann C, Du Y, Paprott R, Haftenberger M, Rathmann W, Scheidt-Nave C. Temporal changes in the prevalence of diagnosed diabetes, undiagnosed diabetes and prediabetes: findings from the German Health Interview and Examination Surveys in 1997-1999 and 2008-2011. Diabet Med 2015 Oct 26. doi: 10.1111/dme.13008.

4. Lange C, Jentsch F, Allen J, Hoebel J, Kratz AL, von der Lippe E, Müters S, Schmich P, Thelen J, Wetzstein M, Fuchs J, Ziese T. Data Resource Profile: German Health Update (GEDA)--the health interview survey for adults in Germany. Int J Epidemiol. 2015; 44:442-450.

5. Wienecke A, Barnes B, Lampert T, Kraywinkel K. Changes in cancer incidence attributable to tobacco smoking in Germany, 1999-2008. Int J Cancer. 2014; 134:682-691.

## S6 Different approaches in estimating the burden of communicable diseases using the examples of the healthcare associated infections and influenza

### Udo Buchholz, Matthias an der Heiden, Tim Eckmanns, Sebastian Haller

#### Department for Infectious Disease Epidemiology, Robert Koch Institute, Berlin, Germany

##### **Correspondence:** Udo Buchholz (BuchholzU@rki.de) – Department for Infectious Disease Epidemiology, Robert Koch Institute, Berlin, Germany

**Outcome-Trees as a first step to estimate the burden of Healthcare Associated Infections**

Assessing the burden of Healthcare Associated Infections (HAI) is challenging. So far published estimates do not fully disclose the actual burden of disease. Outcome-trees with transitional probabilities for mortality and sequelae adjusted for comorbidities were developed to allow disability-adjusted life years (DALY) calculations in an incidence based approach.

Selected HAI (urinary tract infection, primary blood stream infection (neonatal and non -neonatal sepsis [1]), Clostridium difficile infection, pneumonia and lower respiratory tract infections, surgical site infection (joint replacement (hip and knee), coronary artery bypass graft)) were used. Further, results of systematic reviews were included to develop disease specific outcome trees. They reflect the attributable intensive care unit length of stay inclusive of short term complications, attributable mortality and long term complication such as cognitive impairment, posttraumatic stress disease, physical impairment, and chronic renal failure. A protocol was developed to calculate evidence based transitional probabilities adjusted for comorbidities and attributable mortality for HAIs. To calculate the DALYs incidence data may now be entered into the model. So far the role of HAIs is underestimated in GBD and thus also the actual burden of infectious diseases.

**Using non-specific data to estimate the specific burden of disease of influenza in Germany**

The Burden of Communicable Diseases in Europe (BCoDE) project developed a methodology called the pathogen- and incidence-based approach to estimate the burden of infectious diseases [2]. For influenza in Germany, multiplication factors and the burden to be estimated may vary substantially from year to year. Therefore, an alternative approach was developed, estimating the year-specific burden (impact of influenza on each level of the clinical outcome/provider pyramid) based on year-specific data. Sensitive syndromic indicators (e.g. acute respiratory infections, hospitalization due to any respiratory disease and mortality due to any cause) will be included.

The weekly number of confirmed influenza specimens was used as a proxy variable for the season specific course of influenza inside a generalized additive model to reconstruct the course of medically attended acute respiratory infections (MAARI) in Germany. From the same data the type/subtype distribution was inferred to estimate a (sub) type specific number of MAARI infections associated to influenza. First results show that the amount of acute respiratory infections in primary care could be estimated reliably. The approach to estimate/model the burden of influenza on a yearly basis from surveillance and health system data seems feasible and should provide trustworthy and comprehensive year-specific estimates.

**References**

1. Haller S, Deindl P, Cassini A, Suetens C, Zingg W, Abu Sin M, Velasco E, Weiss B, Ducomble T, Sixtensson M, Eckmanns T, Harder T. Neurological sequelae of healthcare-associated sepsis in very-low-birthweight infants: Umbrella review and evidence-based outcome tree. Euro Surveill. 2016;21(8):pii = 30143. DOI: http://dx.doi.org/10.2807/1560-7917.ES.2016.21.8.30143

2. Mangen MJ, Plass D, Havelaar AH et al.. The pathogen- and incidence-based DALY approach: an appropriate [corrected] methodology for estimating the burden of infectious diseases. PLoS One. 2013;8(11):e79740

## S7 Behavioral and environmental attributable risk estimation

### Mohammad Hossein Forouzanfar (forouzan@uw.edu)

#### Institute for Health Metrics and Evaluation (IHME), Seattle, USA

Knowing the overall burden of disease, it is also important to identify which risk factors can be made responsible and where intervention and prevention measures can help to reduce the burden. Risk factor burden estimation directly informs policy makers how to control the burden of diseases in different countries based on the national or, if available, local risk profiles. Environmental and behavioral risk factors cause diseases either directly or through mediated risks such as metabolic risks, for example high systolic blood pressure, high cholesterol and high fasting plasma glucose. This presentation focuses on some environmental and behavioral risks factors that are more relevant to developed countries, including Germany. Data sources for estimating the exposure of risk factors, the disease outcomes of the risk factors and effect sizes were discussed and the methods of exposure estimation for several risks was explained (ambient particulate matter pollution, hand washing, smoking and second hand smoke, diet (14 individual risks including sodium), alcohol consumption, drug use, low physical activity, unsafe sex, childhood sexual abuse, interpersonal violence, and occupational risk factors). Some specific aspects such as risk factor aggregation and mediation are also discussed briefly [1]. In general, the risk factors covered by the GBD-Study were selected based on their relevance for the global population. Nonetheless, countries may have specific risk factor profiles requiring to consider additional risk factors. It can therefore be recommended to conduct national assessments of the disease burden including risk factors that are of importance for specific countries. The current results of the GBD 2013 assessment clearly show, that about 60 % of the overall disease burden measured in DALYs cannot be attributed to any risk factor covered by the GBD study. This however, shows the general need to increase the scope of risk factors. An inclusion of further evidence on risk-outcome-pairs may enable further possibilities to foster measures to further decrease the disease burden.

**References**

1. GBD 2013 Risk Factors Collaborators (2015) Global, regional, and national comparative risk assessment of 79 behavioral, environmental and occupational, and metabolic risks or clusters of risks in 188 countries, 1990–2013: a systematic analysis for the Global Burden of Disease Study 2013. Lancet. 386(10010);2287–2323.

## S8 Environmental Burden of Disease (EBD) in Germany – past achievements and future perspectives

### Dietrich Plass, Myriam Tobollik, Dagmar Kallweit, Dirk Wintermeyer

#### German Environment Agency, Berlin, Germany

##### **Correspondence:** Dietrich Plass (dietrich.plass@uba.de) – German Environment Agency

The EBD concept is increasingly used by international and national institutions to identify key environmental drivers of ill-health. In Germany, especially the German Environment Agency funded and carried out research projects to test the feasibility of the EBD methods on a national scale, quantify the EBD and to evaluate the scientific, legal and ethical challenges introduced with the use of this concept. Air pollution, more specifically particulate matter in ambient air, was identified as the hazard by far causing the highest burden in Germany. A time-series analysis starting in 2007 shows that despite a downward trend, particulate matter pollution in 2013 is still linked to about 43,500 premature deaths and 312,000 years of life lost [1]. Further, second-hand-smoke, residential radon, dioxins, environmental noise, ozone and lead were identified as important contributors to the EBD in Germany [2-3]. However, one might overlook further important hazards where scientific evidence is hampered by uncertainty with regard to the causal link between hazards and health outcomes [4].

Another important stumbling block are the disability weights, which are not available for some health effects caused by environmental hazards and despite the claimed transferability of the GBD-weights to other countries, the discussion on disability weights is still ongoing in Germany.

According to the Agency’s commitment to the precautionary principle, there is not only the need for cross-sectional and retrospective analyses of the EBD, but also for prospective forecasts to shed light on the future burden caused by environmental hazards. Such analyses require robust baseline estimates and reliable prognoses on the epidemiology and demography as well as estimated changes of the population’s exposure to environmental risk factors. These aspects will be covered by a research project, focusing on the EBD in children and adolescents in Germany. As disease burden caused by environmental hazards often follows long lag-periods this project is designed to quantify the current and potential future health burden in a probabilistic framework [5].

Implications for international collaboration

Even though the strength of evidence for some environmental hazards is currently partially limited, assessments should increasingly factor in hazards of concern such as environmental noise and endocrine disrupting chemicals. Despite the importance of air pollution, where data quantity and quality are rapidly increasing, upcoming assessments should also consider new and potential future hazards. In addition to the work on single hazards, collaborations should further foster the estimation of combined effects of environmental risk factors.

**References**

1. Kallweit D (2014) Health risks to the German Population from Fine Particulate Matter. In WHO Collaborating Centre for Air Quality Management and Air Pollution Control No.54-December 2014p. 3-8, https://www.umweltbundesamt.de/sites/default/files/medien/376/dokumente/newsletter_who_cc_air_54.pdf

2. Hornberg C, Claßen T, Steckling N, Samson R, McCall T, Tobollik M, Mekel O, Terschüren C, Schillmöller Z, Popp J, Paetzelt G, Schümann M (2013) Quantifizierung der Auswirkungen verschiedener Umweltbelastungen auf die Gesundheit der Menschen in Deutschland unter Berücksichtigung der bevölkerungsbezogenen Expositionsermittlung - Verteilungsbasierte Analyse gesundheitlicher Auswirkungen von Umwelt-Stressoren, VegAS. Schriftenreihe Umwelt & Gesundheit (01/2013)

3. Hänninen O, Knol AB, Jantunen M, Lim TA, Conrad A, Rappolder M, Carrer P, Fanetti AC, Kim R, Buekers J, Torfs R, Iavarone I, Classen T, Hornberg C, Mekel OCL. Environmental burden of disease in Europe: assessing nine risk factors in six countries. Environmental Health Perspectives. 2014;122(5): 439-446

4. Prüss-Ustün A, Vickers C, Haefliger P, Bertollini R (2011) Knowns and unknowns on burden of disease due to chemicals: a systematic review. Environmental Health; 10(1):9.

5. Plass D, Conrad A, Tobollik M, Wintermeyer D, Gies A. 2015. A first environmental burden of disease assessment for children and adolescents in Germany. In: Abstracts of the 2015 Conference of the International Society of Environmental Epidemiology (ISEE); 30th August – 3rd September 2015; Sao Paulo, Brazil. Abstract 616. Research Triangle Park, NC: Environmental Health Perspectives; http://dx.doi.org/10.1289/ehp.isee2015

## C1 Conclusions of the workshop

### Christa Scheidt-Nave^1^, Thomas Ziese^1^, Judith Fuchs ^1^, Dietrich Plass^2^

#### ^1^Department of Epidemiology and Health Monitoring, Robert Koch Institute, Berlin, Germany; ^2^German Environment Agency, Berlin, Germany

##### **Correspondence:** Christa Scheidt-Nave (Scheidt-NaveC@rki.de) – ^1^Department of Epidemiology and Health Monitoring, Robert Koch Institute, Berlin, Germany

The presentations of the workshop provided deep insights into the concepts and ideas of the GBD framework. They impressively showed the potential of this concept to provide a view on the development of health at the global level as well as at the level of individual countries and regions within countries. Most of the participants of the workshop and especially the WHO representative shared the view that this workshop was a crucial and necessary step for Germany’s engagement in the GBD network and a promising stimulus for a national burden of disease study (NBD).

The experience of Public Health England has nicely shown how sub-national estimates can shed additional light on existing health inequalities at the regional level. Much commitment of local authorities is needed and only a concerted action of national institutions can lead to a success story in terms of a NBD study. Intense and continuous scientific exchange between German public health experts and the GBD representatives could help to expand analyses for Germany and ensure their use for public health policy and practice.

Future research collaborations and the possibility to include burden of disease estimates into national disease surveillance in Germany were discussed with respect to international and national challenges for population health and public health policy.

Overall discussion topics were challenges in the regional variation of health and health care, including estimates of disease prevalence and incidence, the proportion between diagnosed and undiagnosed health conditions. Further, a particular challenge with respect to an ageing population, turned out to be functional capacities, particularly how to consider innovations in diagnostic criteria, and the operationalisation of multimorbidity.

The workshop brought up many ideas of how to improve data collection systems and how use of already available data sources might help to improve the current estimates. National burden of disease surveillance is considered being not only a mission, but a dynamic process with a considerable return on investment and of important value for public health in Germany.

The next step for Germany is to build up a burden of disease collaboration network between universities, institutes and federal as well as regional ministries. Further, it is also envisaged to create an international network of countries who already have conducted a national burden of disease study or who are currently running or planning an assessment. This network is meant as platform to share and discuss methods, results and future public health challenges.

